# Structural Investigations and Binding Mechanisms of Oseltamivir Drug Resistance Conferred by the E119V Mutation in Influenza H7N9 Virus

**DOI:** 10.3390/molecules27144376

**Published:** 2022-07-08

**Authors:** Samuel C. Ugbaja, Sphamandla E. Mtambo, Aganze G. Mushebenge, Patrick Appiah-Kubi, Bahijjahtu H. Abubakar, Mthobisi L. Ntuli, Hezekiel M. Kumalo

**Affiliations:** 1 Drug Research and Innovation Unit, Discipline of Medical Biochemistry, School of Laboratory Medicine and Medical Science, University of KwaZulu-Natal, Durban 4000, South Africa; sphamtambo@gmail.com (S.E.M.); aganzedar@gmail.com (A.G.M.); appiahpat@gmail.com (P.A.-K.); 2 Renewable Energy Programme, Federal Ministry of Environment, 444 Aguiyi Ironsi Way, Maitama, Abuja 900271, Nigeria; bahijjah@yahoo.com; 3 Department of Mathematics, Faculty of Applied Science, Durban University of Technology, Durban 4000, South Africa; mthobisin2@dut.ac.za

**Keywords:** oseltamivir, MD simulations, mutation, neuraminidase, H7N9, influenza, virus, hemagglutinin, wildtype, E119V-mutant

## Abstract

The use of vaccinations and antiviral medications have gained popularity in the therapeutic management of avian influenza H7N9 virus lately. Antiviral medicines are more popular due to being readily available. The presence of the neuraminidase protein in the avian influenza H7N9 virus and its critical role in the cleavage of sialic acid have made it a target drug in the development of influenza virus drugs. Generally, the neuraminidase proteins have common conserved amino acid residues and any mutation that occurs around or within these conserved residues affects the susceptibility and replicability of the influenza H7N9 virus. Herein, we investigated the interatomic and intermolecular dynamic impacts of the experimentally reported E119V mutation on the oseltamivir resistance of the influenza H7N9 virus. We extensively employed molecular dynamic (MD) simulations and subsequent post-MD analyses to investigate the binding mechanisms of oseltamivir-neuraminidase wildtype and E119V mutant complexes. The results revealed that the oseltamivir-wildtype complex was more thermodynamically stable than the oseltamivir-E119V mutant complex. Oseltamivir exhibited a greater binding affinity for wildtype (−15.46 ± 0.23 kcal/mol) relative to the E119V mutant (−11.72 ± 0.21 kcal/mol). The decrease in binding affinity (−3.74 kcal/mol) was consistent with RMSD, RMSF, SASA, PCA, and hydrogen bonding profiles, confirming that the E119V mutation conferred lower conformational stability and weaker protein–ligand interactions. The findings of this oseltamivir-E119V mutation may further assist in the design of compounds to overcome E119V mutation in the treatment of influenza H7N9 virus patients.

## 1. Introduction

The influenza virus has been a burning global concern due to its seasonal occurrences and pandemic nature. It is currently one of the major deadly respiratory diseases. The upsurge in influenza has continued to negatively affect the economic productivity of most temperate and tropical regions where its impacts are prevalent. The influenza virus has been broadly classified as Orthomyxoviridae, having three commonly known types: A, B, and C. The virus is characteristically composed of three membrane proteins: neuraminidase, matrix protein (M2), and hemagglutinin. The presence of neuraminidase and hemagglutinin distinguishes types A and B and makes them potential therapeutic targets [1,2,3,4,5]. Furthermore, other potential pandemic influenza virus subtypes such as H5N1, H7N9, and H9N2 exist [6].

**Figure 1:**
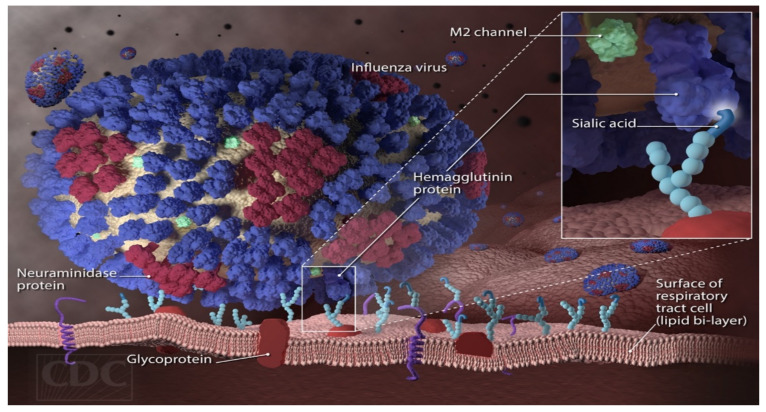
Diagram of the influenza virus’s HA surface proteins binding on the sialic acid receptors of the human cell [10].

For effective pandemic management and therapeutic drug development in the treatment of the avian influenza H7N9 virus, an insight into its mechanism of transmission and replicability is essential. The virus enters through the upper region of the respiratory tract and binds to the α-2,6-link receptors [7,8]. The presence of hemagglutinin (HA) protein allows the entrance of the avian H7N9 virus into the human cell, while the neuraminidase protein enables the exit of the entrapped virus to the other cells of the body. The hemagglutinin has three subunits; one of them binds to the sialic acid receptor of the human cell and subsequently results in its cleavage. This endocytic cleaving allows the entrance of the H7N9 virus into the human cell. The cleavage generates hydrophobic fusion peptides, which are buried in the three subunits. The endocytic vesicles fuse with the lysosomes and lower their pH, which causes the receptor binding to shift backwards. This backward shifting activates the forward movement of the fusion peptide into the vesicular membrane. The H7N9 virus content is then discharged into the cytoplasm, enhancing the chances of the H7N9 virus replication cycle [9]. The avian influenza H7N9 virus attaches to the respiratory tract surface of the sialic acid receptor (Figure 1) in a key lock-like mechanism that results in the host cell’s infection, replication, and subsequent inception of flu [10]. Adults and children with compromised immune systems are more vulnerable to the influenza H7N9 virus. The virus is commonly transmitted in humans through aerosols and droplets of the affected persons when they cough or sneeze in the open. The widely known symptoms of influenza are sore throat, catarrh, cough, muscle pains, feverish conditions, constant headache, and general body fatigue [11].

The first human case of avian influenza H7N9 virus was recorded in March 2013 in China [11]. The world health organisation (WHO) estimated about one hundred and thirty human infections barely four months after the first incidence in March 2013 [12]. According to the WHO, a total of one hundred and six people (in China) were infected with the avian H7N9 virus by January 2017 [12]. The mortality of an estimated two hundred and fifty thousand to five hundred thousand people globally is associated with the influenza virus [13,14].

The neuraminidase cleavage of sialic acid results in releasing the trapped H7N9 virus from the cell. Consequently, when the H7N9 virus is prevented from leaving the cell, it deprives the virus of accessing the necessary resources to replicate, thereby directly inhibiting its replicability and subsequent transmission [15,16]. Over the years, vaccines and antiviral drugs have been major treatment options against the influenza virus [17]; antiviral drugs, which are readily available, are preferred over vaccines, whose development takes a longer [18]. Consequently, because of the presence of neuraminidase proteins in the avian influenza H7N9 virus, the design and development of neuraminidase inhibitors (NAIs) have lately gained popularity in the fight against the deadly virus. Hitherto, the food and drug administration has approved oseltamivir, laminamivir, zanamivir, and, lately, intravenous peramivir [19,20].

**Figure 2:**
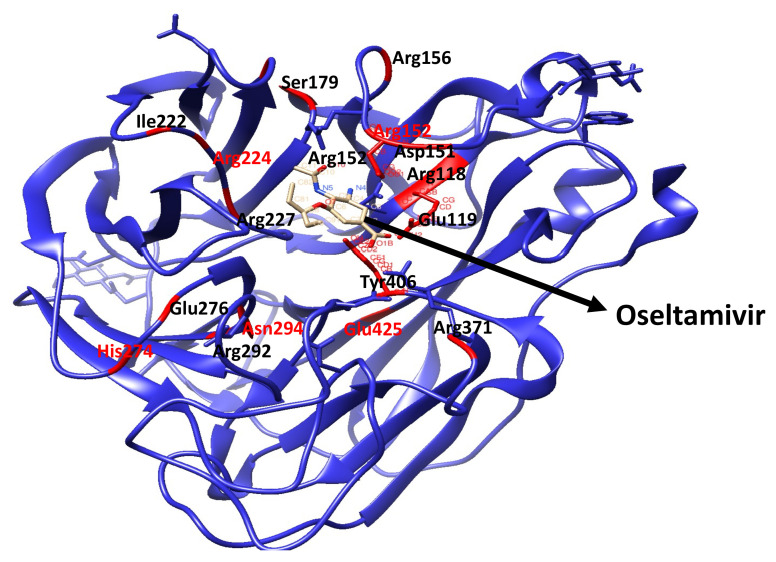
3-D structure of the oseltamivir-neuraminidase complex. The conserved active site and binding cavity supporting enzyme framework residues are indicated in the red ribbon.

Neuraminidase proteins share common characteristic conserved residues within the active site region, which are directly connected to the sialic acid, such as Arg118 (R118), Asp 151 (D151), Arg152 (R152), Arg224 (R224), Glu276 (E276), Arg292 (R292), Arg371 (R371), Tyr406 (Y406) (Figure 2). Furthermore, other residues such as Glu119 (E119), Arg156 (R156), Trp178 (W178), Ser179 (S179), Asp198 (D198), Ile222 (I222), Glu227 (R227), His274 (H274), Glu227 (R227), Asn294 (N294), Glu425 (R425) are considered as binding cavity supporting enzyme frameworks [21,22]. Mutation of any of these conserved residues reduces the susceptibility of the influenza H7N9 virus to neuraminidase inhibitors [23]. Tang et al. (2019) also reported the reduction in the susceptibility of the H7N9 virus to NAIs. The study showed that the mutation of neuraminidase E119 to 119V or 119D resulted in a reduction in susceptibility to oseltamivir and zanamivir [24]. Mariana et al. (2006) had earlier reported infrequent detection of oseltamivir resistance in clinical trials with estimated frequencies of 0.4–1 percent (in adults) and 4–8 percent (in children) on and outpatient care basis [25,26]. Mariana et al. also reported oseltamivir-resistant cases that were 18 percent higher in a childrens’ hospital in Japan. The report further revealed that oseltamivir-resistant species contained substituted amino acid residues 119, 292, and 294 [26]. Prevalent resistance to the single treatment of the influenza H7N9 virus with these drugs led to the combination therapy of these drugs. In 2010, Donald et al. performed an experiment on the effects of the combination of oseltamivir and peramivir [27]. The study explored the chemotherapeutic impacts of the oseltamivir-peramivir combination on mice. A combination of oseltamivir and peramivir in a single dose were administered on some mice with the influenza virus for three days. The mice with combined therapy displayed a speedy and improved recovery compared with their counterparts. Further studies on combinatory treatments showed them to be a better treatment option for the influenza H7N9 virus [27]. Herein, we studied the interatomic and intermolecular dynamic impacts of the experimentally reported E119V mutation on the oseltamivir-resistant influenza H7N9 virus. We extensively carried out molecular dynamic (MD) simulations to investigate the binding mechanisms of oseltamivir-neuraminidase wildtype and E119V mutant complexes. We further analysed the post-MD trajectories to unravel the molecular basis of the E119V mutation as this could help in possible future influenza H7N9 virus drug development.

## 2. Results and Discussions

The analyses of the post-molecular dynamic simulations were evaluated in order to perform a thorough investigation and provide insight into the intermolecular and interatomic dynamic changes resulting from the substituted 119V amino acid residues in the complex. The following post-molecular dynamic simulation analyses were carried out: H-bond analysis, root mean square deviation (RMSD), radius of gyration (RoG), solvent accessible surface area (SASA), root mean square fluctuation (RMSF), principal component analysis (PCA), and binding free energy analysis.

### 2.1. Average Root Mean Square Deviation (RMSD)

**Figure 3:**
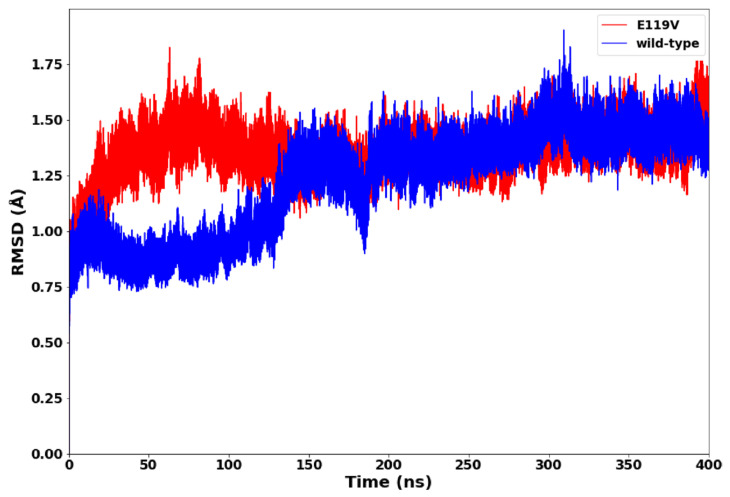
RMSD plot of oseltamivir-wildtype and oseltamivir-E119V mutant complexes.

To analyse the conformational stability of wildtype and mutant proteins and compare their structural dynamic behaviour, the RMSD of backbone C-α atoms was calculated. This result could provide insights into the conformation of the protein throughout the simulation and the RMSD evolutionary trend of the protein. In Figure 3, we present the evaluation of the two systems’ root mean square deviation values. Both complexes demonstrated convergence after 200-ns of the simulation, with the wildtype possessing greater stability (average RMSD = 1.22 Å). In contrast, the high RMSD values in the mutant complex (average RMSD = 1.35 Å) suggested greater mobility of the backbone C-α atoms and less protein stability. Therefore, the E119V mutant complex exhibited a less stable structural conformation due to the mutation.

### 2.2. Root Mean Square Fluctuation (RMSF)

**Figure 4:**
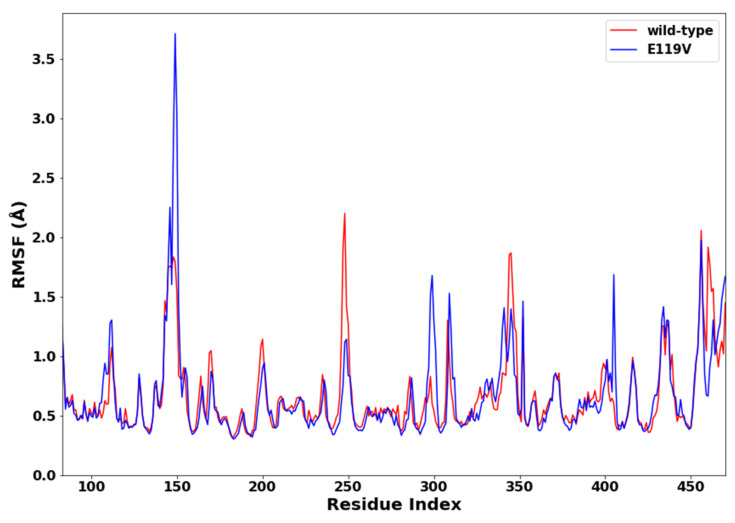
RMSF plot of oseltamivir-wildtype and oseltamivir-E119V mutant complexes.

To indicate differences in flexibility among residues within a system and gain insight into how the mutation impacted the overall residue conformational flexibility, the C-α root mean square fluctuations of the wild type and E119V mutant simulations were calculated. Figure 4 depicts the RMSF calculated for all C-α residues to study the structure’s flexibility. Fluctuations can indirectly alter protein conformation and affect its dynamics, resulting ultimately in reduced functionality. The wildtype and mutant complexes nearly exhibited similar patterns of residue mobility. However, the mutant complex primarily showed higher fluctuations, predominantly between 100 and 150 residues, while the wildtype complex maintained relatively lower fluctuations. As a result, the Val 119 mutation reduced the stability of the oseltamivir-mutant complex.

### 2.3. Radius of Gyration (RoG)

**Figure 5:**
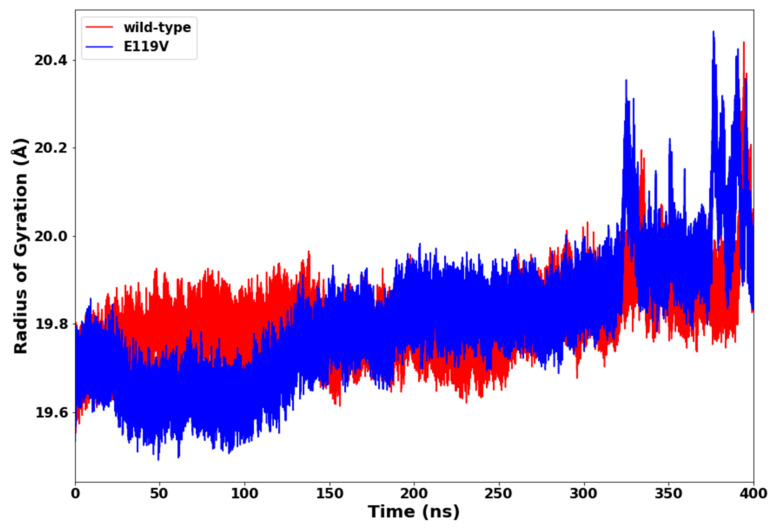
Radius of gyration plot of oseltamivir-wildtype and oseltamivir-E119V mutant complexes.

The radius of gyration is an effective method for determining the compactness of a protein structure. The average radius of gyration of the wild type and the mutant oseltamivir receptor complex were observed to be 19.80 Å and 19.81 Å, respectively which, suggested that there was no significant change in the overall protein compactness (Figure 5).

### 2.4. Solvent Accessible Surface Area (SASA)

**Figure 6:**
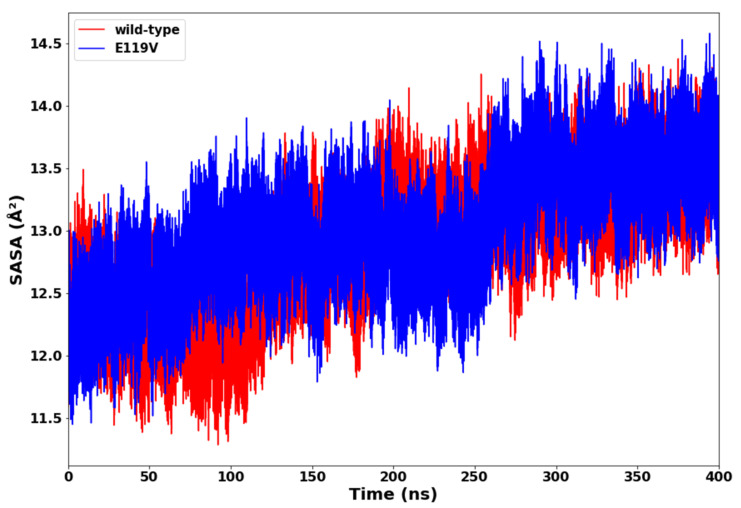
SASA plot of oseltamivir-wildtype and oseltamivir-E119V mutant complexes.

The surface properties of a protein largely determines the interactions between proteins and ligands. Hence, it is crucial to understand how structural deviations affect the solvent-accessible surface area (SASA). The SASA is the amount of surface area available to interact with other ligands, proteins, or solvents. Figure 6 shows the plot of SASA versus time for the wildtype and mutant complexes. The wildtype complex maintained a relatively lower SASA with an average value of 12,875.39 Å^2^, whereas the SASA values for the E119V mutant were relatively higher with an average value of 13,000.89 Å^2^ throughout the simulation. These results indicated that the mutant was thermodynamically unstable, and thereby exposed more proteins to water molecules. An E119V mutation, therefore, reduced the stability of the oseltamivir-mutant complex.

### 2.5. Hydrogen Bond Network Profile

The hydrogen bond percentage (%) occupancy and average distance (Å) between the oseltamivir and active site residues were monitored throughout the 400-ns simulation times (Table 1). Arg 118, Glu 119, Arg 152, Asn 222, Glu 278, and Arg 371 were identified as the primary residues that maintained hydrogen bonding between neuraminidase and oseltamivir with a percentage occupancy above 5%. Hence, the interaction between oseltamivir and these primary residues significantly influenced oseltamivir’s effectiveness.

The wildtype Glu 119-oseltamivir complex showed hydrogen bonding with a percentage occupancy of 5.84%, while the mutant Val 119-oseltamivir complex did not show a hydrogen bond percentage occupancy. This suggested that the E119V mutation induced a loss of hydrogen bond interactions between oseltamivir and Val 119, which might have reduced the binding affinity. The wildtype residues Glu 278 and Asn 222 were involved in hydrogen bonds with oseltamivir with a percentage occupancy of 19.66% and 13.52%, respectively, which were observed to be absent in the mutant. Similarly, a hydrogen bond interaction was formed between Arg118 and oseltamivir in the mutant complex (18.39%) but was absent in the wildtype. The wildtype Arg 152-oseltamivir complex (10.10%) also indicated a lower hydrogen bond occupancy compared to the mutant Arg 152–oseltamivir complex occupancy of 30.40%. The wildtype Arg 371-oseltamivir complex (10.96%) also showed a similar hydrogen bond occupancy to the mutant Arg 371-oseltamivir complex (11.89%). According to these results, the mutant active site’s amino acid residues showed a reduction in the number of hydrogen bond interactions with oseltamivir than the wild type. Consequently, strong interactions of wildtype amino acids with oseltamivir may be vital for higher affinity binding and thermodynamic stability of the oseltamivir-wildtype complex.

### 2.6. Principal Component Analysis (PCA)

**Figure 7:**
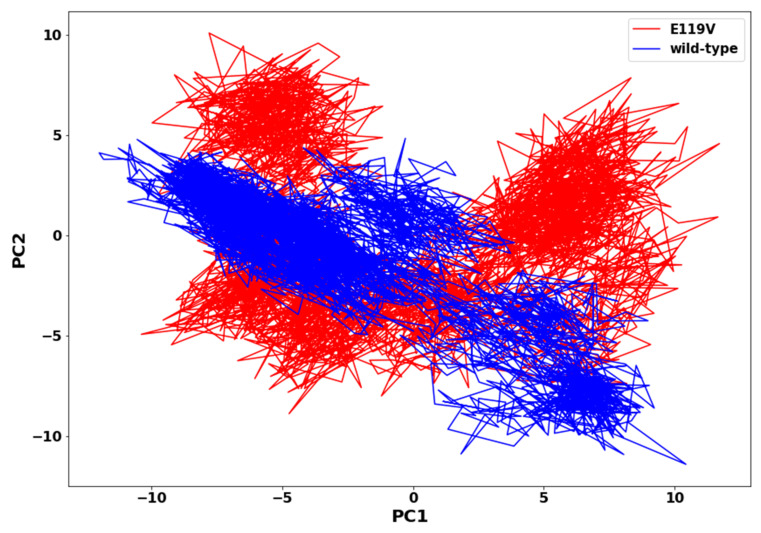
PCA scatter plot projection of PC1 and PC2 for oseltamivir-wildtype and oseltamivir-E119V mutant complex conformations.

Collective motions of proteins are directly related to protein stability and, consequently, are associated with protein function. The principal component analysis provided insight into the overall collective motion of each protein atom in the biomolecular system. The first two principal components (PC1 and PC2) of each atom were computed and projected onto a two-dimensional (2D) subspace for the wildtype and mutant oseltamivir complexes (Figure 7). The E119V mutant structure was observed to cover more area and was more scattered, indicating an uncorrelated variation in dynamics of motion. Thus, the E119V mutant complexed structure displayed a more scattered type of collective motion than the wildtype complexed structure. Therefore, the higher flexibility in the mutant complex compared to the wildtype negatively affected oseltamivir binding interactions.

**Figure 8:**
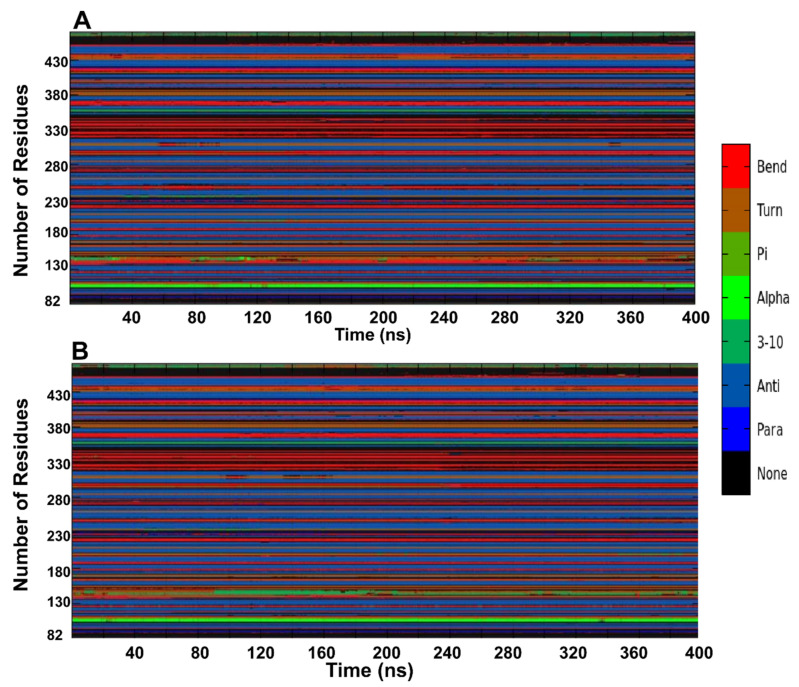
Alterations in the protein secondary structure for wild (**A**) and mutant (**B**) as a function of the 400 ns simulation time.

To further investigate the impact of the E119V mutation, the definition of secondary structure of protein analysis (DSSP analysis) [28] was applied to determine the protein secondary structural changes caused by the mutation. Coils, α-helix, turns, bends, and β-sheets were found in both wild and mutant proteins during simulation. However, the mutant protein showed a minimal decrease in bends forming residues after 160 ns compared to the wild protein (Figure 8). This conformational drift from bends to α-helix forms in the mutant protein after 160 ns within residues 140–160 was due to the amino acid substitution in the mutant structure, which might substantiate the observed less stable, more flexible, and less compact mutant structure.

### 2.7. MM/GBSA Binding Free Energy Calculation

The MM/GBSA method was used to calculate the binding free energy contributions for both oseltamivir-wildtype and oseltamivir-E119V mutant complexes over the 400-ns simulation time, as shown in Table 2.

The calculated total binding energy (ΔG_bind_) for the oseltamivir-wildtype complex was −15.46 ± 0.23 kcal/mol, while for the oseltamivir-E119V mutant complex it was −11.72 ±0.21 kcal/mol. The reduction in binding affinity (−3.74 kcal/mol) might be attributed to the thermodynamic instability of the mutant complex, which resulted in impaired binding interactions and, thus, had the potential to reduce the potency of oseltamivir. The calculated polar solvation energy (ΔG_pol_) of the wildtype complex (36.19 ± 0.76 kcal/mol) was slightly higher than that of the E119V mutant complex (35.80 ± 0.60 kcal/mol). Electrostatic forces (−29.45 ± 0.75 kcal/mol) and Van der Waals forces (−19.26 ± 0.21 kcal/mol) played a significant role in the total binding energies of the wildtype complex compared to the mutant. This might be due to the presence of hydrophobic interactions between amino acid residues in the binding site, which stabilised the conformation of the wildtype complex. These calculations were supported by experimental data showing the E119V mutation increased resistance to oseltamivir 90.77 fold [24] and 88.03 fold [23].

### 2.8. Per-Residue Energy Decomposition

**Figure 9:**
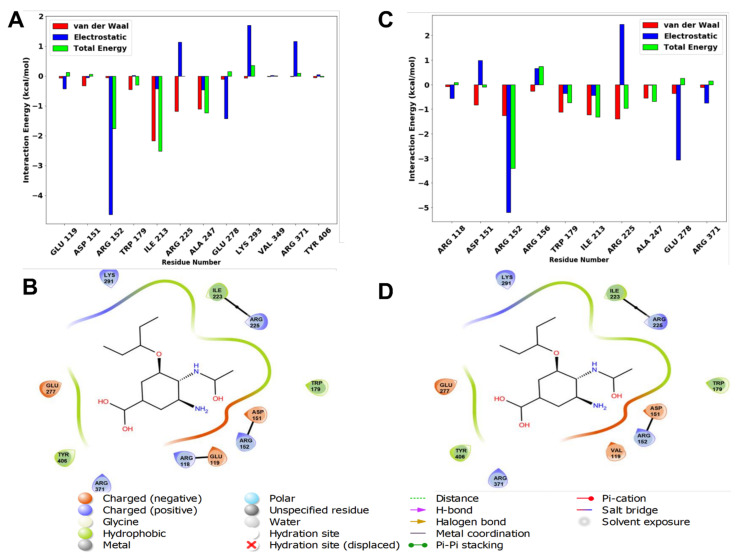
The per-residue energy decomposition analysis of oseltamivir-wildtype (**A**) and oseltamivir-E119V mutant (**C**) complexes, as well as residues interacting at the binding site (**B**,**D**), respectively.

To gain a deeper insight into the amino acid residues that are important for ligand-protein interactions, MM/GBSA was applied. The energy decomposition of protein-ligand interactions per-residue of both the wildtype and E119V mutant complexes is shown in Figure 9.

Figure 9A showed that the wildtype Glu 119 complex had a low residual energy contribution. In contrast, the mutant Glu 119 complex displayed no energy contribution with oseltamivir. This could be due to the E119V mutation destabilising the conformation of the protein-ligand complex at the binding site. As evident in Figure 9A,C, Arg 152 exhibited higher electrostatic energy contributions in both complexes, −4.81 kcal/mol (wildtype) and −5.16 kcal/mol (mutant). Additionally, another prominent electrostatic contributor, Glu 278, was observed to have a more significant effect on binding energy than other residues. These residues are considered critical components of the oseltamivir-neuraminidase binding pocket. The overall higher electrostatic contribution and lower degree of flexibility of the backbone C-α atoms were thought to be accountable for the thermodynamic stability and strong protein-ligand interactions in the wildtype complex.

## 3. Materials and Methods

### 3.1. System Preparation

**Figure 10:**
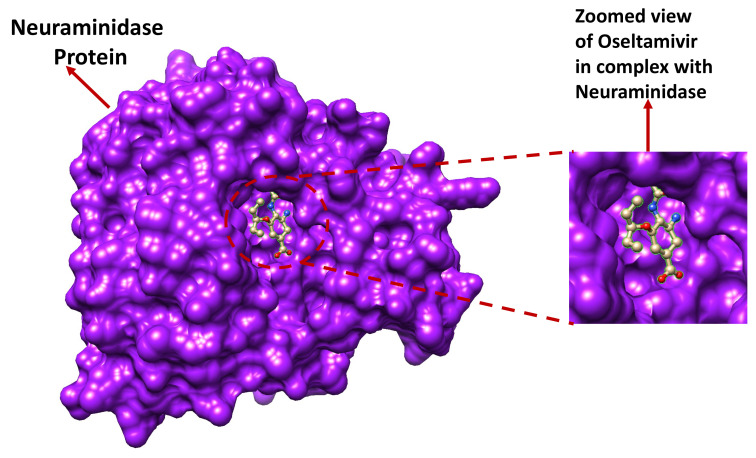
3-D representation of the oseltamivir-neuraminidase complex.

The crystal structural deposit of oseltamivir in complex with the neuraminidase protein (Figure 10), with PDB Code 4MWW, was downloaded from the Research Collaboratory for Structural Bioinformatics (RCSB) protein data bank. The discovery studio software was used in the amino acid substitution (mutation) of Glu119 (E119) to Val119 (119V). Subsequently, receptor and ligand preparations were performed with the UCSF Chimera modelling software [29].

### 3.2. Molecular Dynamic Simulations

A four-hundred-nanosecond (400-ns) MD simulation was carried out on the wildtype and the mutant (E119V) complexes of oseltamivir-neuraminidase. The assisted model building and energy refinement 18 (Amber 18) graphic processing unit (GPU) of PMEMD was implemented in the molecular dynamic simulations of both complexes. The Amber FF14SB was employed in handling the force field related parameters and protein description [30]. The LEAP module implementation of Amber18 was also used to add hydrogen atoms to the receptor and counter-ion for neutralisation of the system [31]. The system was solvated using a TIP3P water box with the atoms located at 8 Å to the water box end. The system utilised periodic boundary conditions while the long-range electrostatics were handled using PMEMD in Amber18 with 12 Å Van der Waals cut off. The initial minimisation was carried out utilising a restrained potential of 500 kcal/mol/Å^2^ in 1000 steepest descent steps and 1000 conjugate gradient steps on the solute. Subsequently, a 1000 step unrestrained conjugate gradient minimisation was performed for the entire system. The system was heated gradually from zero kelvin to three hundred kelvin using a NVT canonical ensemble and a harmonic restraint of 5 kcal/mol/Å^2^ for the solute atoms with a one picosecond random collision frequency. An unrestrained equilibration of the system using NPT ensemble at 1 bar and 300 K was performed. Subsequently, an MD simulation production run of two hundred and fifty nanoseconds was performed with an isothermal isobaric (NPT) ensemble and a Berendsen barostat [32]. The atom coordinates of the systems were saved at intervals of 2 ps, and the trajectories were analysed. Post molecular dynamic simulation MD analyses were carried out using Amber18 module CPPTRAJ. The visualisation of the molecular structures was performed by UCSF Chimera molecular modelling software and the plotting of graphs and charts with origin software [33].

### 3.3. Root Mean Standard Deviation (RMSD)

Root mean square deviation was used to measure the displacement of atoms or atom groups within a given MD simulation [34]. For a set of *N* atoms, the time averaged RMSD was calculated as follows:

(1) $$RMSD=\sqrt{\frac{\sum_{i=1}^{N}di^{2}}{N}}$$

where *di* is the distance between atom *i* and the mean position of the *N* equivalent atoms [35,36].

### 3.4. Root Mean Standard Fluctuation (RMSF)

Root mean square fluctuation (*RMSF*) is a calculation that estimates the fluctuation of atoms or atom groups relative to their average position within a measured MD trajectory [34]. The *RMSF* analysis was calculated using the following equation:

(2) $$sRMSF_{i}=\frac{\left(RSMSF_{i}-RMSF\right)}{\sigma \left(RMSF\right)}$$

where *RMSF_i_* represents the *RMSF* of the *i^th^* residue, from which the average *RMSF* is subtracted. Then the *RMSF*’s standard deviation [σ(*RMSF*)] is divided by *RMSF* to yield the resultant standardised *RMSF* (*sRMSF_i_*) [35,36].

### 3.5. Radius of Gyration (RoG)

The equilibrium conformation of a protein structure within a given trajectory in MD simulation is referred to as RoG. The RoG describes the RMSD of atoms from the common centre of gravity of a given enzyme molecule [37]. The RoG was determined using the following equation:

(3) $$r^{2}g=\frac{\sum_{i=0}^{n}W_{i}{\left(r_{i}-r^{-}\right)}^{2}}{\sum_{i=1}^{n}W_{i}}$$

where *r_i_* denotes the position of the *i^th^* atom and r the centre mass atom *i*. The mean value was determined by taking RoG values over the number of frames in a given trajectory.

### 3.6. Principal Component Analysis (PCA)

Principal component analysis (PCA) was used to obtain collective motion coordinates representing the overall dynamics of each trajectory. The covariance matrix was diagonalised to yield a set of eigenvectors and eigenvalues [38]. The 400-ns MD trajectories of oseltamivir-wildtype and oseltamivir-E119V mutant complexes were stripped of solvent and ions using the PTRAJ module [39] of AMBER 18. Each trajectory of the MD simulation was calculated for the covariance matrix (C-α atoms) between residues i and j [40]. In-house scripts were used to calculate the first two principal components (PC1 and PC2) and generate the covariance matrix. The first two principal components corresponded to the first two eigenvectors of the covariance matrix. The PCA scatter plots were then constructed with Matplotlib [41,42].

### 3.7. Thermodynamic Analysis

The binding free energy calculation involves the endpoint energy analysis and subsequent provision of essential information on the receptor-ligand complex relationship. In an ideal spontaneous reaction with equilibrium states of constant pressure and temperature, receptor-ligand complex occurs when the system’s change in Gibbs free energies (ΔG) is negative. Contingent upon the fact that the receptor-ligand association is relative to the magnitude of the −Δ*G*, therefore, it is suggested that the stability of any given receptor-ligand complex is controlled by Δ*G* [33,43]. Furthermore, Δ*G* is determined by the initial and final thermodynamic states, irrespective of the pathway connecting these states. The binding free energies of oseltamivir-wildtype and oseltamivir-E119V mutant complexes were analysed using the molecular mechanics/generalized-born surface area (MM/GBSA) method. The binding free energy was therefore summarised by the following equations:

ΔG_bind_ = G_complex_ − G_receptor_ – G_ligand_ (4)

ΔG_bind_ = E_gas_ + G_sol_ – TΔS (5)

E_gas_ = E_int_ + E_vdW_ + E_ele_ (6)

G_sol_ = G_GB_ + G_SA_ (7)

G_SA_ = γSASA (8)

where E_gas_ is the gas phase energy; Eint is the internal energy; Eele the is the electrostatic (Coulomb) energy, while Evdw is the Van der Waals energy. The gas phase energy was estimated directly from the FF14SB force field terms. The solvation free energy was decomposed into polar and non-polar states. The polar salvation G_GB_ contribution was evaluated by solving the GB equation, whereas the non-polar solvation contribution, G_SA_, was determined from the solvent-accessible surface area (SASA) assessed by using a water probe radius of 1.4 angstroms. T represents the temperature, while S is the total solute entropy. The individual amino acid contributions to the total binding free energy of the oseltamivir-neuraminidase complexes were calculated by the interaction energy decomposition analysis per residue using the Amber18 molecular mechanics/generalised-born surface area binding free energy method [33,43]. A total of 1000 snapshots were evenly extracted over the last 300-ns of the MD trajectories at intervals of 300 ps.

## 4. Conclusions

The structural and dynamical behaviours of oseltamivir-wildtype and oseltamivir-E119V mutant complexes were investigated through various computational approaches in order to understand the impact of the E119V mutation on resistance to oseltamivir. The oseltamivir-wildtype complex demonstrated low residue flexibility and higher conformational stability based on the RMSD, RMSF, SASA, and PCA profiles. The study showed that the E119V mutation affected oseltamivir’s optimal orientation within the active site, its overall conformation, and the hydrogen bond interaction network between proteins and ligands. The decrease in binding affinity (−3.74 kcal/mol) might be caused by the thermodynamic instability of the mutant complex, leading to impaired binding interactions, which reduced oseltamivir’s potency. In the wildtype complex, the higher electrostatic energy contributions and low residue flexibility were thought to be responsible for the thermodynamic stability and strong protein-ligand interactions. The findings of this study provided a basis for investigating other mutations that might affect the efficacy of oseltamivir against the avian influenza H7N9 virus.

## Figures and Tables

**Table 1:** Percentage (%) occupancy and average distance (Å) between the oseltamivir (OSELTA) and primary active site residues.

Complexes	Acceptor	DonorH	Donor	Frames	Percentage Occupancy	Average Distance
	GLU278@OE1	OSELTA@H1	OSELTA@O1B	78632	19.66	2.64
	ASN222@O	OSELTA@H1	OSELTA@O1B	54091	13.52	2.77
Wild-type	OSELTA@O1A	ARG371@HH22	ARG371@NH2	43836	10.96	2.84
	OSELTA@O10	ARG152@HH22	ARG152@NH2	40403	10.10	2.82
	GLU119@OE2	OSELTA@H4	OSELTA@N4	23366	5.84	2.86
	OSELTA@O10	ARG152@HH12	ARG152@NH1	121.609	30.40	2.81
E119V	OSELTA@O1A	ARG118@HH12	ARG118@NH1	73546	18.39	2.84
	OSELTA@O1A	ARG371@HH12	ARG371@NH1	47549	11.89	2.87

**Table 2:** MM/GBSA binding free energy contributions for oseltamivir-wildtype and oseltamivir-E119V mutant complexes.

Complexes	ΔG_vdv_	ΔE_ele_	ΔE_gas_	ΔG_pol_	ΔG_nonpol_	ΔG_sol_	ΔE_bind_
Wild-type	−19.26 ± 0.21	−29.45 ± 0.75	−48.71 ± 0.92	36.19 ± 0.76	−2.94 ± 0.04	33.25 ± 0.72	−15.46 ± 0.23
E119V	−17.57 ± 0.26	−27.33 ± 0.53	−44.90 ± 0.75	35.80 ± 0.60	−2.62 ± 0.04	33.18 ± 0.56	−11.72 ± 0.21

## Data Availability

The data presented in this study are available on request from the corresponding author.

## Sample Availability

Not available.
